# The diagnostic performance of machine learning based on resting-state functional magnetic resonance imaging data for major depressive disorders: a systematic review and meta-analysis

**DOI:** 10.3389/fnins.2023.1174080

**Published:** 2023-09-22

**Authors:** Yanjing Chen, Wei Zhao, Sijie Yi, Jun Liu

**Affiliations:** ^1^Department of Radiology, Second Xiangya Hospital, Central South University, Changsha, Hunan, China; ^2^Clinical Research Center for Medical Imaging in Hunan Province, Changsha, Hunan, China

**Keywords:** depression, machine learning, functional connectivity, functional MRI, support vector machine

## Abstract

**Objective:**

Machine learning (ML) has been widely used to detect and evaluate major depressive disorder (MDD) using neuroimaging data, i.e., resting-state functional magnetic resonance imaging (rs-fMRI). However, the diagnostic efficiency is unknown. The aim of the study is to conduct an updated meta-analysis to evaluate the diagnostic performance of ML based on rs-fMRI data for MDD.

**Methods:**

English databases were searched for relevant studies. The Quality Assessment of Diagnostic Accuracy Studies (QUADAS-2) was used to assess the methodological quality of the included studies. A random-effects meta-analytic model was implemented to investigate the diagnostic efficiency, including sensitivity, specificity, diagnostic odds ratio (DOR), and area under the curve (AUC). Regression meta-analysis and subgroup analysis were performed to investigate the cause of heterogeneity.

**Results:**

Thirty-one studies were included in this meta-analysis. The pooled sensitivity, specificity, DOR, and AUC with 95% confidence intervals were 0.80 (0.75, 0.83), 0.83 (0.74, 0.82), 14.00 (9, 22.00), and 0.86 (0.83, 0.89), respectively. Substantial heterogeneity was observed among the studies included. The meta-regression showed that the leave-one-out cross-validation (loocv) (sensitivity: *p* < 0.01, specificity: *p* < 0.001), graph theory (sensitivity: *p* < 0.05, specificity: *p* < 0.01), *n* > 100 (sensitivity: *p* < 0.001, specificity: *p* < 0.001), simens equipment (sensitivity: *p* < 0.01, specificity: *p* < 0.001), 3.0T field strength (Sensitivity: *p* < 0.001, specificity: *p* = 0.04), and Beck Depression Inventory (BDI) (sensitivity: *p* = 0.04, specificity: *p* = 0.06) might be the sources of heterogeneity. Furthermore, the subgroup analysis showed that the sample size (*n* > 100: sensitivity: 0.71, specificity: 0.72, *n* < 100: sensitivity: 0.81, specificity: 0.79), the different levels of disease evaluated by the Hamilton Depression Rating Scale (HDRS/HAMD) (mild vs. moderate vs. severe: sensitivity: 0.52 vs. 0.86 vs. 0.89, specificity: 0.62 vs. 0.78 vs. 0.82, respectively), the depression scales in patients with comparable levels of severity. (BDI vs. HDRS/HAMD: sensitivity: 0.86 vs. 0.87, specificity: 0.78 vs. 0.80, respectively), and the features (graph vs. functional connectivity: sensitivity: 0.84 vs. 0.86, specificity: 0.76 vs. 0.78, respectively) selected might be the causes of heterogeneity.

**Conclusion:**

ML showed high accuracy for the automatic diagnosis of MDD. Future studies are warranted to promote the potential use of these classification algorithms in clinical settings.

## Introduction

Major depressive disorder (MDD) is a global leading cause of emotional disorders with a high recurrence and suicide rate (Mccarron et al., 2021). It can seriously affect the physical and mental health of patients and has brought a huge burden to society (Osler et al., 2015). Even though the complex interactions between genetics and the environment are involved in the cause of the disease, a large number of underlying biomarkers still dominate its development. Up to now, the diagnosis of depression is still based on psychiatrists’ assessments and interviews, which is subjective to some extent. Moreover, the applicability of a depression scale like the Hamilton Depression Scale (Hamilton, 1960) (HDRS/HAMD), which is used to assess the outpatient, is questionable (Uher et al., 2008). The subjective scale may contribute to delayed diagnosis, and then affect prognosis (Pearson et al., 1999; Almeida, 2014; Park and Zarate, 2019). Therefore, objective biomarkers are urgently needed to diagnose MDD.

Neuroimaging, which is widely used in clinical practice, has been proven to provide several objective biomarkers for the diagnosis of MDD. Resting-state functional magnetic resonance imaging (rs-fMRI) is one of the functional neuroimaging modalities that is rapidly being utilized to investigate the brain biomarkers of psychiatric diseases. In rs-fMRI procedures, the brain’s activity is monitored by the changes in blood oxygenation (Le Bihan et al., 1995), which alters the magnetic properties of the blood and then produces the signals. The rs-fMRI has been used as an alternative strategy for the early screening of depression (Kassraian-Fard et al., 2016). Many encouraging biomarkers obtained from rs-fMRI, i.e., amplitude of low frequency fluctuation (ALFF), regional homogeneity (ReHo), and functional connectivity (FC), are used to diagnose MDD; however, the analysis procedure is complex and the results are varied with low specificity. In this context, research on MDD using rs-fMRI is nowadays mostly focused on exploring the biological mechanisms behind depression, and can hardly apply into clinical diagnosis and prediction.

Since the introduction of artificial intelligence, there has been a multitude of studies that have used machine learning to diagnose diseases and predict the efficacy of treatment (Kumar et al., 2022). Apart from saving a certain amount of time and the cost of manual evaluation (Valenstein et al., 2001), a combination of machine learning and rs-fMRI can diagnose mental diseases precisely (Khanna et al., 2015) and is essential to the clinical application of objective neuroimaging in mental diseases (Fernandes et al., 2017; Zhang X. et al., 2020). As a multivariate model, machine learning is able to tap into the complex relationship between brain changes and depression symptoms deeply, which most simple rs-fMRI analysis approaches cannot do (Haynes, 2015). For example, Chekroud et al. (2016) found that clinical non-symptomatic features incorporated in machine learning can be very helpful in predicting the treatment outcome of MDD. Chen et al. (2022) discovered that the diagnostic value of imaging metrics can be partly realized with machine learning. An issue with the current use of rs-fMRI to differentiate psychiatric disorders is that changes may involve the same brain regions for different disorders which induces low specificity. Sha et al. (2018) constructed different support vector machine (SVM) models depending on the same frontal striatal dysfunction to differentiate obsessive–compulsive disorder (OCD) from schizophrenia. Unsupervised learning is used to capture features with higher specificity in samples of a large size, which will be more likely to explain the neural basis of depression (Rutledge et al., 2019).

Despite the many benefits described above, machine learning studies on depression diagnosis using rs-fMRI data are few and immature. Due to the small sample sizes used in previous studies and relying solely on single training and validation methods, The diagnostic performance is not reliable. It is also challenging to select proper features from the high-dimensional rs-fMRI data. As is known, changes in functional connectivity can reflect the ability of information transfer between brain regions. With the introduction of topology, the synchronous changes in the brain have attracted attention, and brain network indicators can reflect the overall or local changes of brain neurons, which is of great significance for the regulation of certain behavioral traits. Therefore, special feature selection is crucial for diagnosing depression and reflecting depressive behavioral traits. According to past findings that used brain anatomy data in machine learning, the key to optimizing the diagnostic model is applying the appropriate subjects rather than modifying the algorithm (De Martino et al., 2008). No studies have ever reported the characteristics and quantitative effects of the sample on the model.

Therefore, our objective is to use meta-analysis to evaluate the diagnostic performance of ML based on rs-fMRI data for MDD and further explore the underlying relevant variables.

## Materials and methods

We conducted and report this meta-analysis based on the PRISMA (Preferred Reporting Items for Systematic Reviews and Meta-Analyses) guidelines (Moher et al., 2009).

### Literature search

Electronic databases including the PubMed, Embase, Web of Science, and Cochrane Library databases were searched by two observers independently to identify studies. The searches were performed on 23 February 2022. The search terms consisted of the following terms: ((“Machine Learning” [Mesh]) OR (“machine learning”) OR (“ML”)) AND ((“resting-state functional magnetic resonance”) OR (“rs-fMRI scans”) OR (“rs-fMRI”)) AND ((“Major Depressive Disorders”[Mesh]) AND (“Depression”) AND (“MDD”)); ((“Artificial Intelligence” [Mesh]) OR (“artificial intelligence”) OR (“AI”)) AND ((“Functional Magnetic Resonance Imaging” [Mesh]) OR (“fMRI scans”) OR (“fMRI”) OR (“functional MRI”) OR (“functional magnetic resonance imaging”)) AND ((“Major Depressive Disorders”[Mesh]) AND (“depression”) AND (“MDD”)).

### Study selection

The titles and abstracts of potentially relevant studies were additionally screened by two reviewers [a doctoral student with 2 years of post-graduate experience in medical image analysis (XX) and a radiologist in the fourth year of training (LB)].

All of the studies were selected according to the following criteria: (a) original research studies; (b) patients with depression were enrolled who were assessed using scales; (c) rs-fMRI was applied to classify MDD and HC using ML; and (d) data were sufficient to reconstruct the 2 × 2 contingency table to estimate the sensitivity and specificity of the diagnosis.

Studies were excluded if: (a) they were reviews, editorials, abstracts, or animal studies; and (b) structural magnetic resonance imaging (sMRI) or task-based fMRI (t-fMRI) was applied to classify MDD and HC by ML; and (c) the information needed could not be calculated from the articles.

### Data extraction

Relevant data were extracted from each study, including the names of the authors, year of publication, demographic characteristics of HC and patient groups [group size, age, sex, symptoms as measured by the Hamilton Depression Rating Scale (HDRS/HAMD), the Beck Depression Inventory (Beck et al., 1961) (BDI), or the Patient Health Questionnaire-9 (Kroenke et al., 2001) (PHQ-9), magnetic field strength, training and validation methods, and features selected].

For each study, the true positive (TP), false positive (FP), false negative (FN), and true negative (TN) values were extracted, and a pairwise (2 × 2) contingency table was created.

### Data quality assessment

The Quality Assessment of Diagnostic Accuracy Studies (QUADAS-2) was used to assess the methodological quality of the included studies and the risk of bias at the study level (Whiting et al., 2011), which consisted of: (a) patient selection; (b) index test; (c) reference standard; and (d) flow and timing.

### Statistical analysis

This meta-analysis was conducted using Stata software, version 16.0, and Review Manager software, version 5.3. The predictive accuracy was quantified using pooled sensitivity, specificity, diagnostic odds ratio (DOR), positive likelihood ratio (PLR), and negative likelihood ratio (NLR) with 95% confidence intervals (CIs). The summary receiver operating characteristic curve (SROC) and area under the curve (AUC) were used to summarize the diagnostic accuracy. Q and *I*^2^ were calculated to estimate the heterogeneity among the studies included in this meta-analysis. Pooling and effect size were evaluated using a random-effects model, indicating that estimating the distribution of true effects between studies considered heterogeneity (Deeks et al., 2005). Meta-regression analysis was conducted to further investigate the cause of the heterogeneity. Subgroup analysis was performed to examine the potential effects of different demographic factors, ML algorithms, and types of training and validation.

### Publication bias

The publication bias was assessed using Deek’s funnel plot asymmetry test, where a *p* value <0.05 suggested a potential publication bias. Deek’s funnel plot asymmetry test was performed using Stata 16.0.

## Results

### Literature search

**Figure 1:**
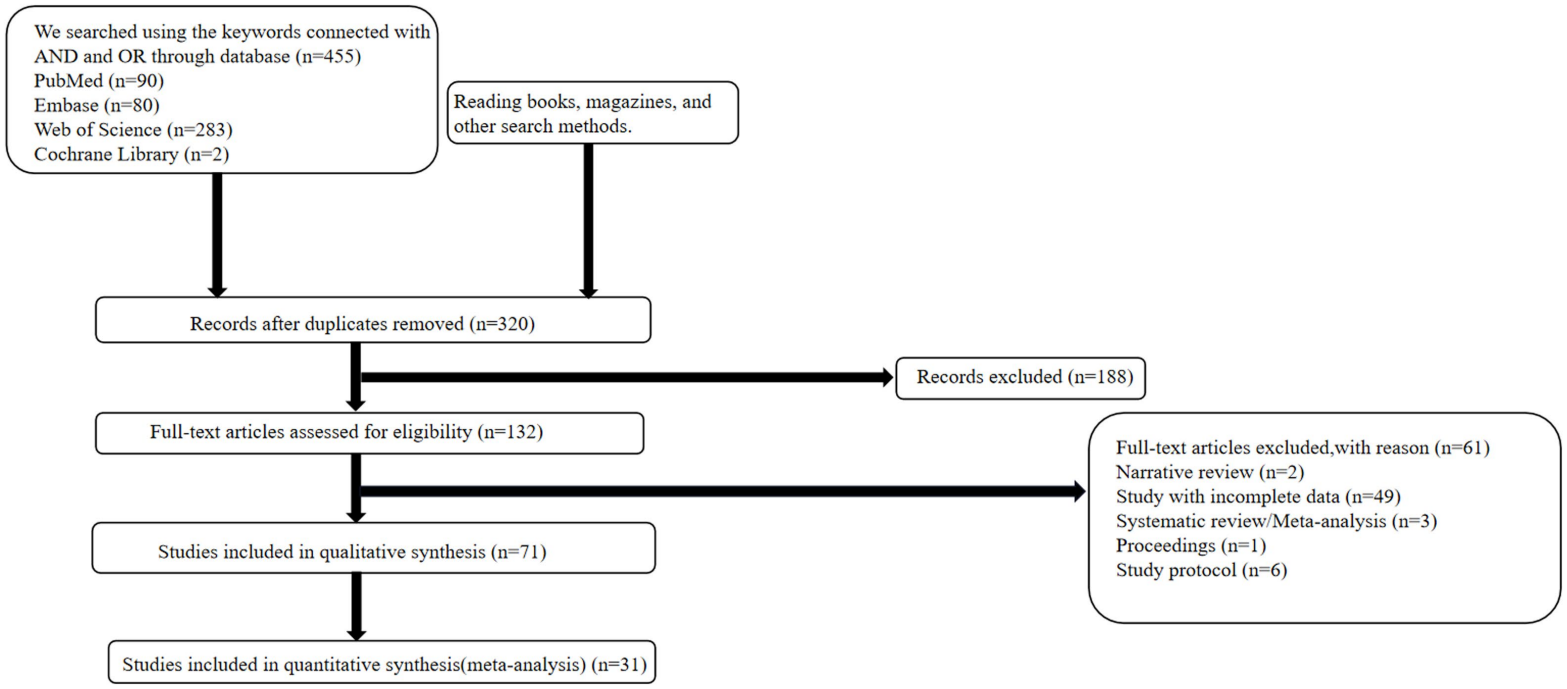
Flow diagram of the study selection for meta-analysis.

The complete literature search flowchart is presented in Figure 1. According to the search strategy described above, 455 potentially eligible citations were identified. After screening titles and abstracts, we excluded 135 studies for duplication and 249 studies for non-relevant abstracts or publication types. Finally, after revision, 40 articles were excluded, leaving 31 articles for inclusion in the meta-analysis.

### Data quality assessment

The quality assessment of the included studies using the QUADAS-2 checklist is presented in Supplementary Figure S1. Overall, generally, the data quality was considered acceptable.

### Study characteristics

The characteristics of the included studies are summarized in Table 1. The 31 studies included in this review had 2,699 participants where ML models were used to diagnose MDD. All of the studies used retrospectively collected data. Of these models, the ML algorithm comprised different types of models; most of them were support vector machine models, (SVM) (*n* = 16). Some articles had multicenter samples referring to several kinds of models such as linear discriminant analysis (LDA) and extreme gradient boosting (Xgboot) (*n* = 5). The HDRS/HAMD was used in 17 studies, and the BDI/BDIII/PHQ was used in 5 studies. The scores of HDRS/HAMD of current depression can be divided into three severity. Status 18 to 22 ≤22 (*n* = 5), 22 to 24 (*n* = 4), or > 24 (*n* = 5) indicated presenting symptoms were mild, moderate, or severe, respectively. In 31 articles, different kinds of features derived from resting-state were used, including functional connectivity (*n* = 25), graph theory (*n* = 5), and ReHo (*n* = 1). The sample size for MDD of four studies was larger than 100 and the remaining ones (*n* = 27) were smaller than 100. Eleven studies employed five-fold or ten-fold cross-validation as the test method, and twenty studies employed the leave-one-out cross-validation method. Exclude articles that cannot calculate diagnostic indicators information, there were 16 studies that used 3 T MRI scanners and 6 studies used 1.5 T MRI scanners. Siemens MRI equipment (*n* = 11) was used more than GE Healthcare MRI equipment (*n* = 5). The information about the neuropsychological estimates can be seen in Supplementary Table S1.

**Table 1:** rs-fMRI-based machine learning characteristics of studies included in the systematic review.

	Author	N (MDD)	Sex (MDD, M/F)	Age (MDD)	Equipment	Machine learning method	Features	Model validation	Evaluation scale
31	Chun et al. (2020)	262	100/162	32.91 ± 11.07	3.0 T	SVM, XGboot, RF, CNN	FNC	Ten-fold cross-validation	NA
30	Nakano et al. (2020)	163	NA	44.10 ± 12.20	3.0 T	SVM, RF	FC	Seven-fold cross-validation	NA
29	Zhang et al. (2021)	60	9/22	50.50 ± 11.20	NA	JSD	Graph theory	NA	NA
28	Guo et al. (2017)	38	15/23	28.40 ± 9.68	3.0 T Siemens	SVM	(High order) Graph theory	NA	HAMD: 22.8
27	Zhao et al. (2020)	269	105/164	32.80 ± 10.60	3.0 T	GAN, SVM, AdaBoot	FNC	Ten-fold cross-validation	HDRS: 18.3
26	Zeng et al. (2012)	24	8/16	31.83 ± 10.99	1.5 T GE	SVM	Kendall rank correlation coefficient	LOOCV	HDRS: 26.2
25	Wei et al. (2013)	20	10/10	34.30 ± 8.20	3.0 T Siemens	SVM	Hurst exponent FC	LOOCV	HDRS: 25.8
24	Yu et al. (2013)	19	11/8	26.65 ± 7.62	1.5 T GE	SVM	FC	LOOCV	HRSD: 25.4
23	Drysdale et al. (2017)	220	NA	NA	NA	SVM	FC	LOOCV	NA
22	Zeng et al. (2014)	24	8/16	31.83 ± 10.99	1.5 T GE	SVM, LDA, MMC	FC	LOOCV	HRSD: 25.4
21	Zheng et al. (2019)	82	29/53	30.84 ± 10.38	3.0 T GE	SVM	Hurst exponent FC	LOOCV	HAMD: 22.46
20	Lord et al. (2012)	22	13/9	34.55	3.0 T Siemens	SVM	Graph theory	NA	HAMD: 15.80
19	Ramasubbu et al. (2016)	18	6/12	38.00 ± 10.00	3.0 GE	SVM	FC	Five-fold cross-validation	NA
18	Sharaev et al. (2018)	25	NA	NA	1.5 T Toshiba	SVM	Graph theory	NA	NA
17	Mousavian et al. (2021)	38	11/27	18.00 ± 15.00	NA	NA	FC	Ten-fold cross-validation	BDI-II: 13.00
16	Jun et al. (2020)	29	8/21	43.79 ± 13.06	3.0 T Siemens	GCN	FC	Ten-fold cross-validation	HDRS: 14.48
15	Jing et al. (2017)	19	NA	34.84 ± 13.58	3.0 T Siemens	SVM	Dynamic FC (DFC)	LOOCV	HAMD: 21.65
14	Yoshida et al. (2017)	58	NA	42.8 ± 11.9	3.0 T GE	KPLS-LDA	FC	LOOCV	BDI-II: 30.90
13	Yan et al. (2020)	0.43	13/30	35.23 ± 11.23	3.0 T GE	SVM	DFC	Ten-fold cross-validation	HAMD: 23.35
12	Guo et al. (2019)	38	15/23	28.4 ± 8.99	3.0 T Siemens	SVM	Graph theory	Ten-fold cross-validation	HAMD: 22.80
11	Bhaumik et al. (2017)	38	9/29	20.97 ± 1.53	3.0 T GE	SVM	FC	LOOCV	HAMD: 2.39
10	Shi et al. (2021)	1,021	336/685	35.52 ± 13.40	NA	Xgboot	FC	Ten-fold cross-validation	HAMD: 21.70
09	Li et al. (2021)	14	6/8	27.38 ± 7.38	3.0 T Siemens	KELM	ReHo	Five-fold cross-validation	PHQ-9: 18.21
08	Jacob et al. (2020)	21	NA	NA	7.0 T	QLA	FC	Five-fold cross-validation	NA
07	Cao et al. (2014)	39	16/23	27.99 ± 7.49	1.5 T Siemens	SVM	FC	LOOCV	HAMD: 24.97
06	Wang et al. (2016)	29	15/14	NA	1.5 T GE	WDDL, SVM	FC	LOOCV	NA
05	Sundermann et al. (2017)	49	13/36	15.62 ± 2.86	NA	SVM	Dynamic FC	LOOCV	BDI-II: 29.00
04	Geng et al. (2018)	24	8/16	51.2 ± 10.6	3.0 T Siemens	SVM, KNN, LR	Effective connectivity	LOOCV	BDI: 32.30
03	Craddock et al. (2010)	20	8/12	43.2 ± 10.8	3.0 T Siemens	SVC	FC	LOOCV	NA
02	Sen et al. (2021)	180	94/86	50.8 ± 7.1	3.0 T	SVM	FC	Ten-fold cross-validation	HAMD: 20.20
01	Patel et al. (2015)	27	7/20	70.20 ± 7.98	3.0 T Siemens	ADTree	FC	LOOCV	HAMD: 20.33

SVM, Support-vector machine; LOOCV, Leave-one-out cross-validation; LDA, Linear discriminant analysis; FC, Functional connectivity; XGBoost, Extreme gradient boosting; RF, Random forests; PLS, Partial least squares regression; ADTree, Accurate, detailed, and automatic modelling of laser-scanned trees; HAMD, Hamilton Depression Scale; BDI, Beck Depression Inventory; PHQ-9, Patient Health Questionnaire-9; KNN, K-Nearest neighbor; SVC, Support vector classification; WDDL, Weighted discriminative dictionary learning; GCN, Graph convolutional network; JSD, Jensen–Shannon Divergence.

### Pooled results

**Figure 2:**
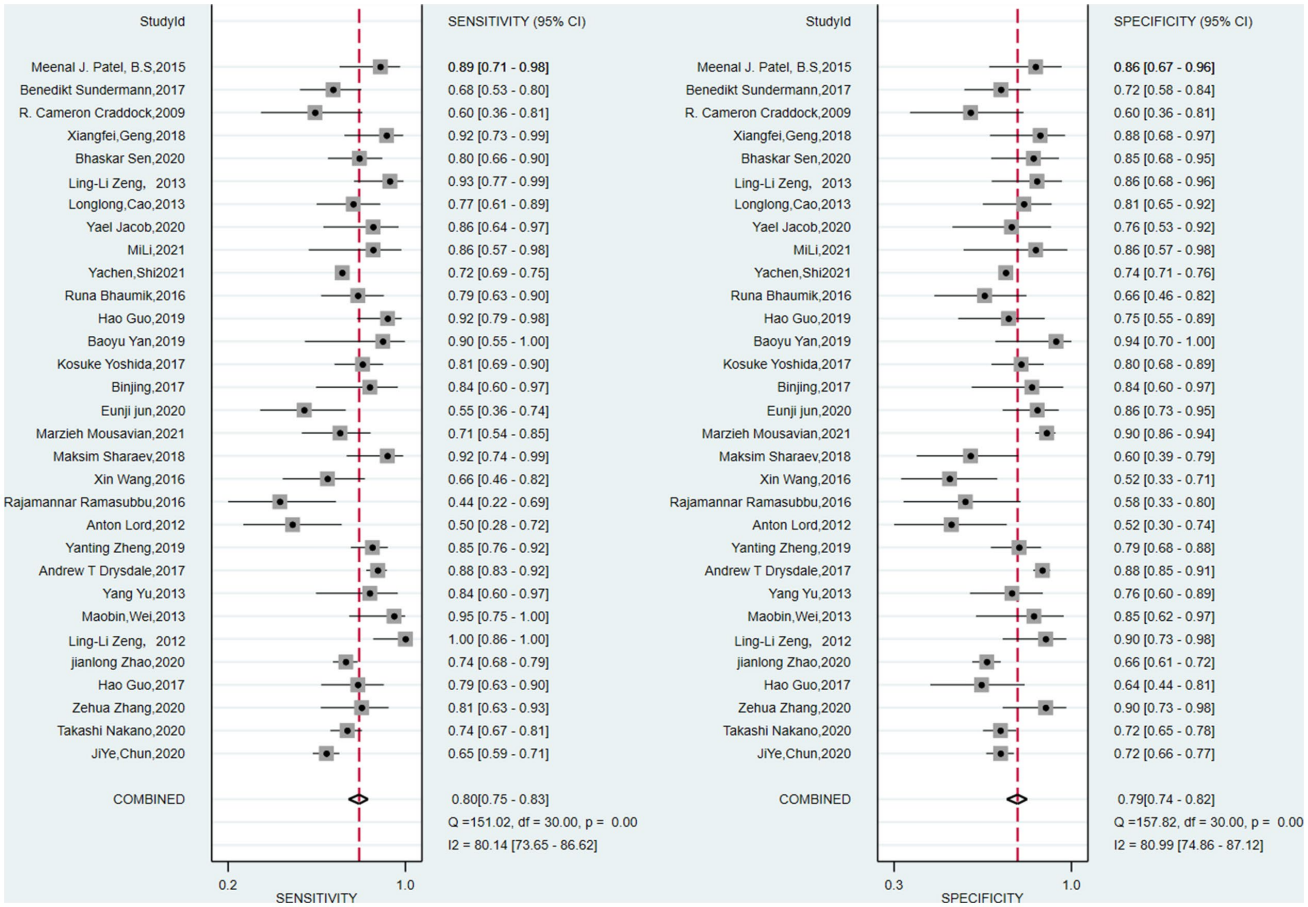
Pooled estimates of sensitivity and specificity of machine learning to differentiate major depressive disorders from healthy controls. On the left represents the annotation for each article, we only use the first name of the first author or the corresponding author.

**Figure 3:**
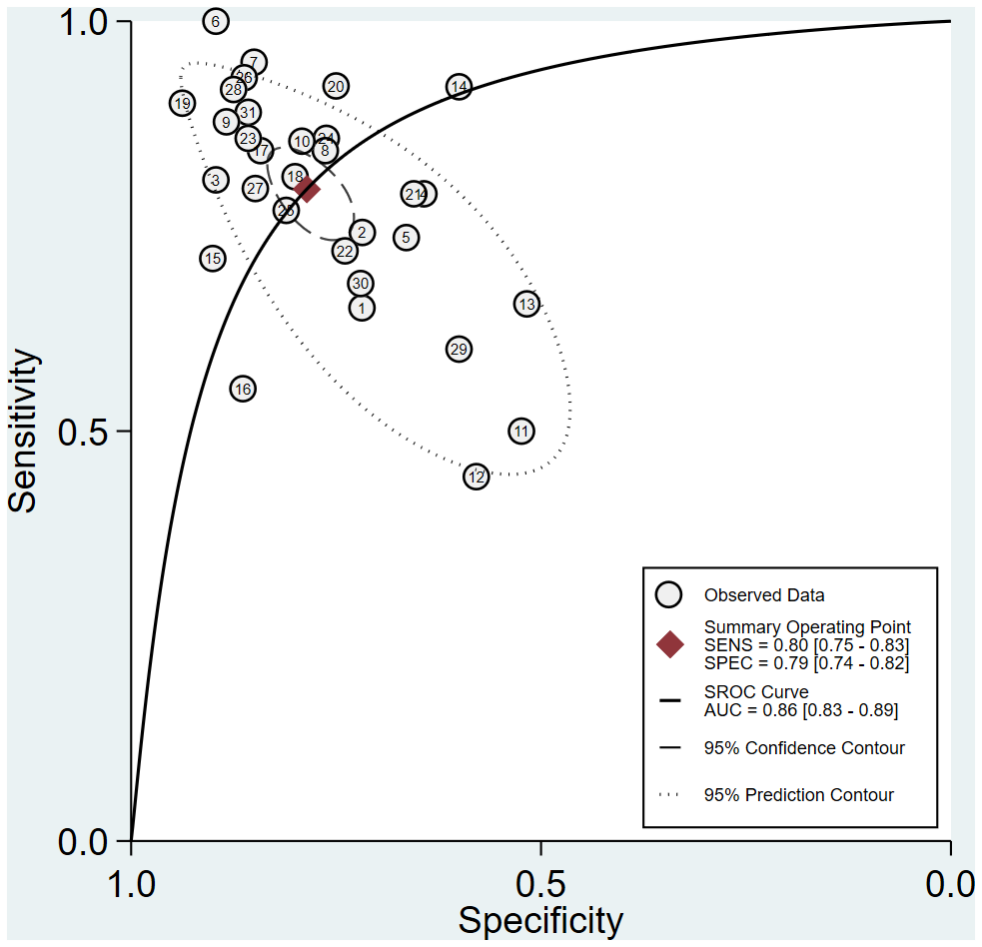
Summary receiver operating characteristic curve (SROC) of the diagnostic performance of ML to distinguish MDD and HC.

The pooled sensitivity and specificity of machine learning for discriminating MDD and HC were 0.80 (95% CI: 0.75 to 0.83) and 0.79 (95% CI: 0.74 to 0.82), respectively. The forest plots are shown in Figure 2. The pooled PLR and NLR were 3.7 (95% CI: 3.0 to 4.6) and 0.26 (95% CI: 0.20 to 0.33), respectively. The DOR was 14 (95% CI: 9 to 22). SROC curve analysis was used to summarize the overall diagnostic accuracy. The AUC was 0.86. The SROC curve is shown in Figure 3. The results demonstrated high diagnostic performance in discriminating MDD from HC.

### Exploration of heterogeneity

**Figure 4:**
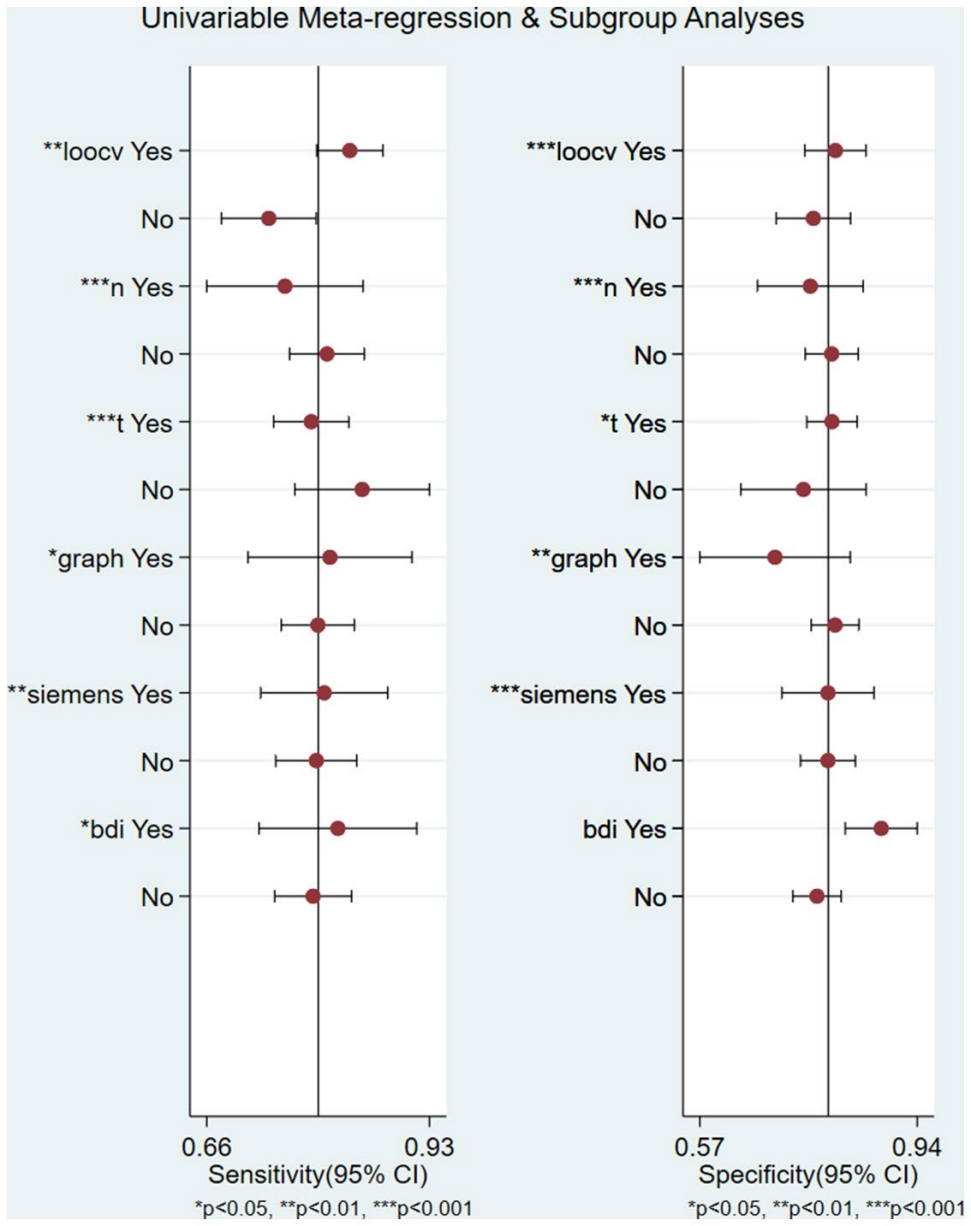
Univariable meta-regression plot of machine learning for the diagnosis of depression in factor of leave-one-out crossvalidation, graph, 3 T field strength, Siemens equipment, and BDI/HDRS scales.

There was significant heterogeneity in sensitivity (*I*^2^ = 80.14%) and specificity (*I*^2^ = 80.99%). Subgroup analysis and meta-analysis were performed by comparing studies with the different variables. Table 2 shows the results of the analysis for subgroups. Studies (*n* = 4) with a large sample size (>100) after excluding one article had a lower specificity (0.71 vs. 0.81) and lower sensitivity (0.72 vs. 0.79) compared with studies (*n* = 26) with a small sample size when the symptoms of the patient were similar. The studies that used graph theory had equal sensitivity (0.86 vs. 0.84) and lower specificity (0.76 vs. 0.78) compared with those (*n* = 8) that used functional connectivity as a feature. Four studies with self-rating scales such as the BDI or PHQ-9 as the evaluation standard had a lower sensitivity (0.86 vs. 0.87) and specificity (0.78 vs. 0.80) than studies (*n* = 4) using the HDRS/HAMD. Meta-regression (Supplementary Figure S2) using modifiers identified in the systematic review was conducted; we found that the leave-one-out cross-validation (sensitivity: *p* < 0.01, specificity: *p* < 0.001), graph theory (sensitivity: *p* < 0.05, specificity: *p* < 0.01), BDI (sensitivity: *p* = 0.04, specificity: 0.06), 3T (sensitivity: *p* < 0.001, specificity: *p* = 0.04), *n* > 100 (sensitivity: *p* < 0.001, specificity: *p* < 0.001), and Siemens equipment (sensitivity: *p* <0.01, specificity: *p* < 0.001) were the sources of heterogeneity (Figure 4).

**Table 2:** Results of pooled estimates of all studies and of different subgroups.

Studies	Number of studies included	Sensitivity (95% CI)	Specificity (95% CI)	PLR	NLR	DOR
All studies						
Overall	31	0.80 (0.75–0.83)	0.79 (0.74–0.82)	3.7 (3.0–4.6)	0.26 (0.20–0.33)	14 (9–22)
Sample size (*n*), patients with severe symptoms:						
*n* > 100	4	0.71 (0.68–0.74)	0.72 (0.68–0.75)	2.5 (2.2–2.8)	0.4 (0.36,0.45)	6 (5–8)
*n* < 100	26	0.81 (0.75–0.86)	0.79 (0.74–0.83)	3.9 (3.0–5.0)	0.24 (0.18–0.32)	16 (10–27)
The scores of different degrees of disease with HDRS (*n* < 100):						
Mild (<22)	5	0.52 (0.39–0.65)	0.62 (0.52–0.71)	1.4 (0.9–2.1)	0.77 (0.53–1.11)	2 (1–4)
Moderate (22–24)	4	0.86 (0.80–0.90)	0.78 (0.71–0.83)	3.8 (2.9–5.1)	0.19 (0.13–0.26)	21 (12–35)
Severe (≥24)	5	0.89 (0.77–0.95)	0.82 (0.75–0.87)	4.9 (3.3–7.2)	0.14 (0.06–0.31)	36 (12–109)
Features selected: graph theory, patients with moderate symptoms:						
Graph theory	4	0.84 (0.77–0.89)	0.76 (0.64–0.85)	3.5 (2.3–5.3)	0.21 (0.14–0.30)	17 (9–32)
Functional connectivity	4	0.86 (0.80–0.90)	0.78 (0.71–0.83)	3.8 (2.9–5.1)	0.19 (0.13–0.26)	21 (12–35)
Different scales: patients with moderate and severe symptoms:						
BDI	4	0.86 (0.80–0.90)	0.78 (0.71–0.83)	4.3 (3.4–5.6)	0.17 (0.11–0.24)	21 (12–35)
HDRS/HAMD	9	0.87 (0.81–0.91)	0.80 (0.75–0.84)	4.3 (3.4–5.6)	0.17 (0.11–0.24)	26 (15–46)

### Publication bias

There was no publication bias based on the Deek’s funnel plot (*p* = 0.07) (Supplementary Figure S2).

### Clinical utility

Using an ML-based model increased the post-test probability to 48% from 20% with a PLR of 4 when the pretest was positive and would reduce the post-test probability to 6% with an NLR of 0.26 when the pretest was negative (Supplementary Figure S3).

## Discussion

Until now, it has been extremely difficult to make accurate diagnoses and predictions in psychiatry. Although rs-fMRI is a widely available tool for psychiatric research, the lack of specificity has prevented it being effectively applied in clinical practice (Casey et al., 2013). Artificial intelligence (AI) has been shown to improve medical diagnosis and assist in building more accurate and realistic models of neural functioning through the analysis of fMRI data (Cohen et al., 2017). The current study provides compelling evidence of the high accuracy of machine learning using rs-fMRI to diagnose depression. Due to the intricacies of psychiatric disorders, the influence of other factors should be considered more carefully in the implementation of subgroup analysis. Our study found that potential confounding factors, including sample size, validation strategy, and disease severity, can impact the construction of reliable and comprehensive models (Claeys et al., 2022).

Regression with sample size as a moderator showed a significant effect on both sensitivity (*p* < 0.001) and specificity (*p* < 0.001). Subgroup analysis showed a large sample size (>100) exhibited lower specificity (0.71 vs. 0.81) and sensitivity (0.72 vs. 0.79) than a small sample size (*n* < 100). This was in line with previous research, which found that small sample size (*N* = 20) accuracies were up to 95%, while the accuracy of medium sample sizes (*N* = 100) were up to 75% (Flint et al., 2021). When there are few data samples and many features, the biased accuracies are typically visible (Simon, 2003). The amount of data is one of the three challenges with applying functional neuroimaging in the era of big data (Rajalakshmi et al., 2018; Li et al., 2019). It has been demonstrated that the amount of data available has a considerably greater impact on model construction than algorithms performance (Hidalgo-Mazzei et al., 2016). The majority of the studies included in our research proposed the model validated on a single site; in contrast, the five articles in our study containing large and multicenter samples employed the model validated on multiple sites. The pipeline can give us a complete view of how to deal with the data through machine learning, such as external validation methods between different sites and the training methods used.

The support vector machine (SVM) algorithm was employed to categorize patients in the vast majority of studies in the present research since it is well recognized in machine learning to handle noisy, correlated characteristics and high-dimensional data sets. It was significant that the articles using large samples used other different classification algorithms, such as Xgboot and LDA, to obtain better diagnostic performance. When there are many more candidate features than cases, decomposition and grouping techniques are the best option for understanding the true neural basis (Khosla et al., 2019). Combining various classifiers, such as the SVM and the logistic regression or the SVM and the linear discriminant analysis, was more effective than using only one to identify MDD (Yan et al., 2020). The size of the dataset and the feature selection technique are two variables that affect the choice of a suitable classifier. Therefore, it is worthwhile to investigate the application further (Pereira et al., 2009). Validation is another crucial element. As the meta-regression showed, different cross-validation methods can lead to different conclusions (sensitivity: *p* = 0.00, specificity: *p* = 0.00). The leave-one-out cross-validation (loocv) is a common approach in which the prediction algorithm is built using all of the training data except for one observation (Tai et al., 2019). Varoquax demonstrated that (Varoquaux et al., 2017), although this technique is good and can strengthen the model structure, it may result in unreliable accuracy when is compared to five-fold or ten-fold cross-validation. If the sample size is limited, cross-validation could cause major statistical errors (Varoquaux, 2017) that cannot be changed by optimizing the model. This training–testing strategy based on complex data sets has been heavily depended upon to improve the accuracy of diagnosis models, and this research anticipated creating a meta-analytical framework for clinical decision-making in psychiatry diagnosis (Iwabuchi et al., 2013).

We also found that studies using Siemens MRI equipment were one of the sources of heterogeneity (sensitivity: *p* < 0.01, specificity: *p* < 0.001). This means different MRI equipment may affect the diagnostic performance. Therefore, prospective studies comparing the two pieces of MRI equipment are necessary to explore the diagnostic performance of rs-fMRI-based diagnosis. However, previous studies have solved the problem of data drift caused by data collection from different sites through algorithm optimization. Gradient matching federated domain adaptation (GM-FDA) is a domain adaptation algorithm which combines the ideas of federated learning and domain adversarial training. This method has been used to solve the problem of poor performance of machine learning models on different devices, especially mobile devices. Zeng et al. effectively applied this method to solve the issue of low generalization ability of previous machine learning models related to neuroimaging and validated it for the diagnosis of depression.

Our study found that the usual features selected in publications were the functional connectivity between different brain regions. They were typically selected using lasso-regularized logistic regression (lasso) or tested using permutation (Zhang R. et al., 2020) because of the large amount and result of overfitting. This meta-regression and sub-analysis showed that the powerful classifying capacity of the topology features derived from graph theory analysis was almost equal to the result of functional connectivity (sensitivity: 0.84 vs. 0.86; specificity: 0.76 vs. 0.78). It can be used to assess the centrality of the brain network (the betweenness centrality, eigenvector centrality, participation coefficient, and within module *z*-score) (Sotero, 2016), as well as integration (characteristic path length and efficiency) and segregation (clustering coefficient and transitivity). Kazeminejad and Sotero (2018) found graph theoretical analysis is more reliable than earlier analysis technique applied and can effectively cancel out the effects of multisite and multi-device MRI sequences. What is more, the data obtained are not too so much that they can also achieve a good training effect (Wang et al., 2017). According to Wang et al. (2017) found that deficiencies in the topological structure underlying emotion processing could help distinguish MDD from other mental disorders. Topological features can be used to display the entire pathological imbalance of brain connections induced by depression (Zhang R. et al., 2020), and there are some discrepancies between the functional and structural topological properties in MDD. Kambeitz et al. (2017) The overall diagnostic efficiency(88% sensitivity, 92% specificity) using DTI as the characteristic is higher than that using rs-fMRI. (85% sensitivity, 83% specificity). The same graph metrics may describe different physiological pathology in structural and functional networks (Xu et al., 2021). Until now, there have not been many resting-state graph theoretical analyses that are worthy of being carried out. Other research also described some novel rs-fMRI analysis methods such as effective connectivity and dynamic functional connection, which can provide more knowledge about the brain (Xiao et al., 2020; Ji et al., 2021). A multimodal MRI connectome study is still a prospective direction. Due to the sample size, we did not perform the sub-analysis of their diagnostic accuracy, which may be another future direction.

A prior work hypothesized that the severity of clinical symptomatology was correlated with the degree of functional and structural brain abnormalities seen in depression (Demenescu et al., 2011; Mwangi et al., 2012). Machine learning had been reported to be able to predict the severity of depression according to functional connectivity features. It is yet unknown how well it can diagnose different degrees of depression (Kessler et al., 2016); the current subgroup study preliminary addressed the limitation of the research by Kambeitz et al. by showing that the accuracy of diagnosing severely unwell subjects was higher than that of diagnosing the moderately and mildly ill (sensitivity: 0.89 vs. 0.86 vs. 0.52; specificity: 0.82 vs. 0.78 vs. 0.62, respectively). We also observed that similar illness states assessed by different depression scales might not correspond to similar brain circumstances. This is one of the drawbacks of using behavioral assessment for psychiatric disorders. In our study, the judgment of BDI-based ML diagnosis accuracy was worse than the HDRS-based ones (sensitivity: 0.86 vs. 0.87, specificity: 0.78 vs. 0.80). There are differences in the assessment of depressive states when using BDI/BDI-II on the same individual, and these differences tend to increase gradually with severity (Furukawa et al., 2019), which involves consistency of various scales (Rabinowitz et al., 2022). When there is not a perfect association, a comparison between them can potentially offer helpful clinical information (Petkova et al., 2000; Targum et al., 2013). The BDI scales could additionally experience the same issues as other self-report scales because scores can easily be exaggerated or minimized under specific circumstances (Pop-Jordanova, 2017). The evaluator’s incorrect interpretation of the rules (Monica et al., 2008) and the subjects’ careless responses may also result in the failure of assessment. This finding convinced us that pure behavioral assessments were easily affected and unreliable. Combining behavioral traits with objective changes in brain function is more persuasive for screening depression. It is feasible to utilize the multivariable property of machine learning to connect depressive scales with functional MRI and develop a practical model, consistent with previous research conclusions (Stoyanov et al., 2019), in the primary time, which is advantageous given the difficulty of translating neuroimaging to clinic application.

### Limitations and future direction

As psychiatric disorders were inherently heterogeneous and the subjects included were complex, we used subgroup analysis to select some potential variables and the *I*^2^ was reduced at the same time. There were still some inconsiderable factors such as antidepressant medication, age, and sex (Liu et al., 2019). The gender and age ratios were consistent with the epidemiology of depression and other relevant data the articles provided were limited or ambiguous, so we were unable to analyze further. In addition, some articles used the same data to test the diagnostic efficacy of different combinations of models. We selected the best results for inclusion in the study, which were in line with the machine learning training guidelines for building models. Negative results were not presented in articles so a publication bias might have occurred.

In our subgroup analysis, we obtained high specificity of machine learning diagnostic performance for feature selection and sample size. This is a crucial point explored in this study. However, uncontrollable factors during this process may still affect machine learning in diagnosing depression, such as cross-site data collection and the selection of preprocessing step parameters. Standardization and streamlining of this part of the process will play a decisive role in providing neurobiological information for the diagnosis of depression using machine learning methods in the future. The limited sample size of severely depressed patients used in previous studies has restricted our exploration of the significance of machine learning selection methods. The lack of early sensitivity markers in the clinic can be addressed to a certain extent through the combination of neuroimaging and machine learning methods. However, this process still requires repeated testing and verification, and the use of large databases can save time and manpower. In the future, the establishment and open availability of large-scale databases can create even greater potential for efficient transformation of resting-state fMRI information using machine learning methods.

From the results of this meta-analysis, We concluded that the sample size had a significant impact on the model’s accuracy; therefore, it is crucial to carry out external validations in larger samples to encourage generalizability. Another direction for the future is the use of multi-modal imaging data to create better models, as it will be more advantageous to include proteomics or genomes while tracking depression in its early stages.

## Conclusion

There is more and more research using machine learning based on rs-fMRI to identify psychiatry like depression. Our work revealed that machine learning may be a reliable technique for differentiating depression from healthy controls on the basis of neural mechanisms after displaying some possible characteristics. It is hoped that this will eventually turn into a controllable instrument.

## Author contributions

YC and WZ were responsible for the original study design. YC was responsible for the original search, identification of relevant manuscript, and initial drafting of the report. YC and SY were responsible for data extraction, risk of bias assessments, and data analysis. WZ and JL critically revised the article. All authors contributed to the article and approved the submitted version.
