# Supplementary material for: The diagnostic performance of machine learning based on resting-state functional magnetic resonance imaging data for major depressive disorders: a systematic review and meta-analysis
Source: Front Neurosci. 2023 Sep 22;17:1174080. doi: 10.3389/fnins.2023.1174080 (PMC10559726; doi:10.3389/fnins.2023.1174080)
Supplement: Supplementary file 1 [file Data_Sheet_1.docx]

Supplementary Material:


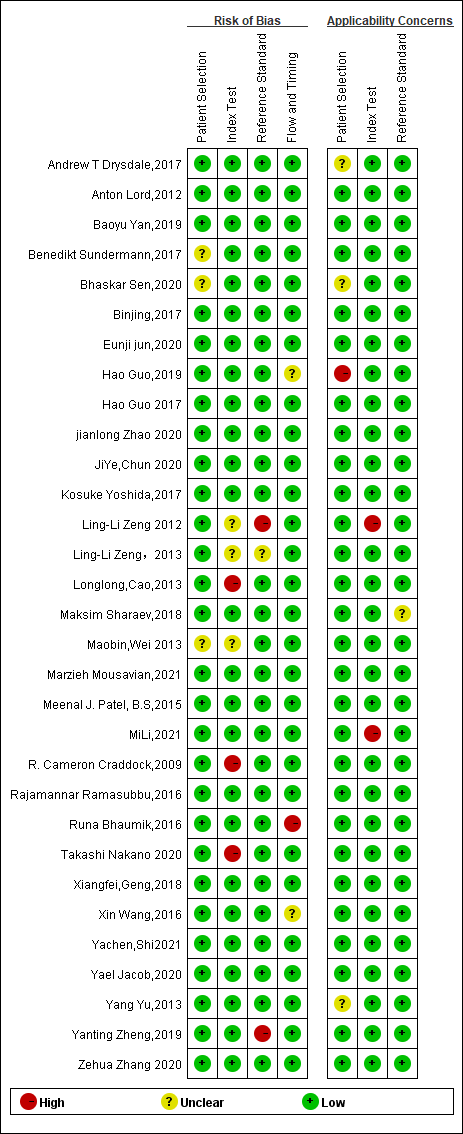


**Figure S1A** The Methodological quality summary using QUADAS-2


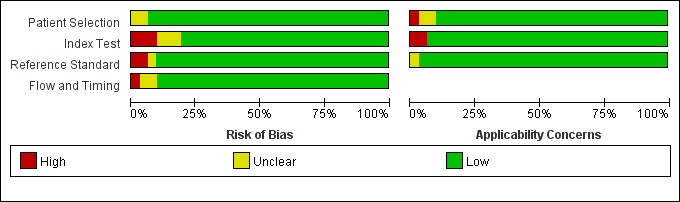


**Figure S1B** risk of bias (left) and concerns for applicability (right) for each included study using QUADAS-2


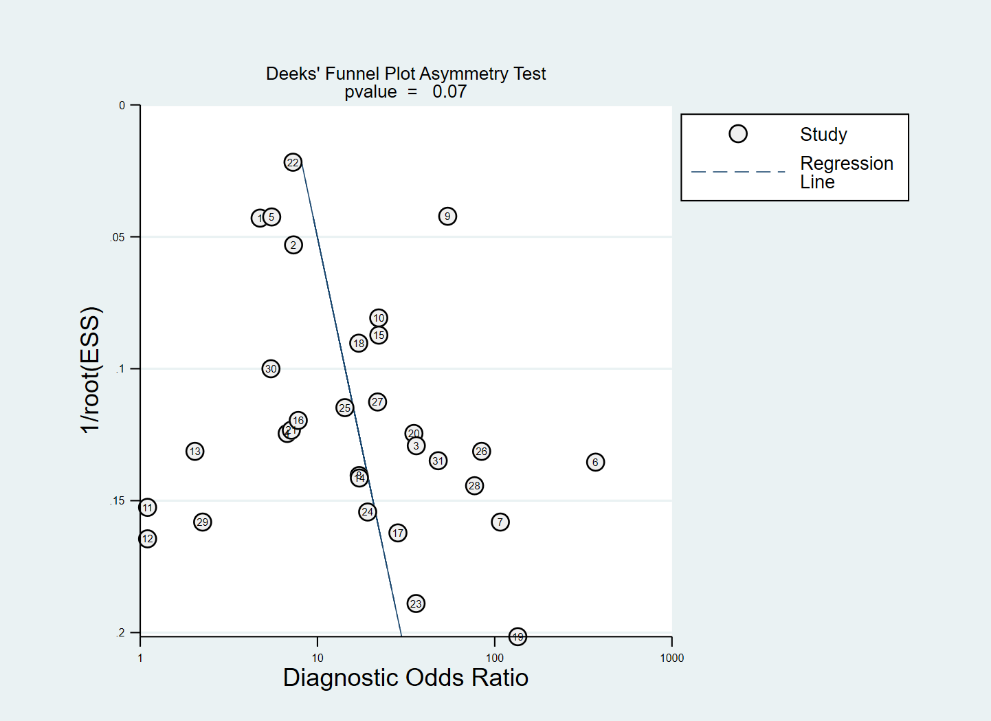


**Figure S2:** Deeks funnel plot shows the likelihood of publication bias is low with a P value of 0.07.ESS, effective sample size.


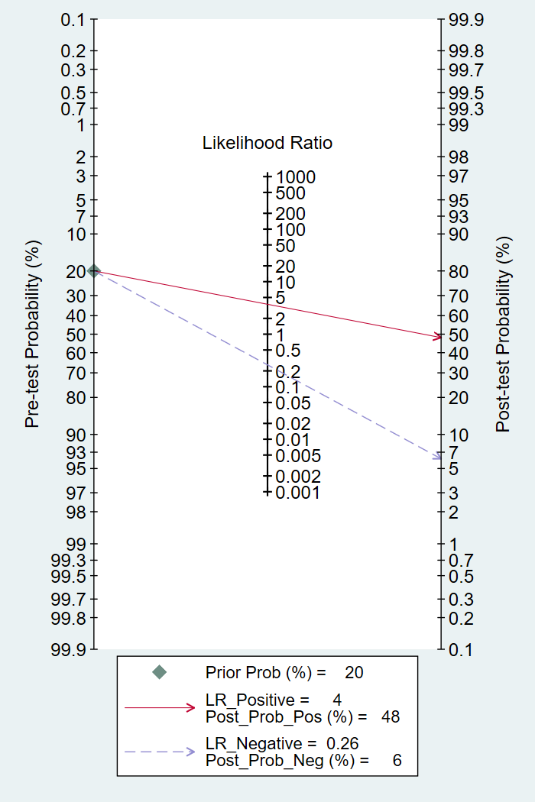


**Figure S3:** Fagan plot of ML-based models of rs-MRI in diagnosing depression.
